# Platelet-Rich Plasma Injections in Chronic Lateral Ankle Instability: A Case Series

**DOI:** 10.3390/biomedicines12050963

**Published:** 2024-04-26

**Authors:** Ivan Medina-Porqueres, Pablo Martin-Garcia, Sofia Sanz-De-Diego, Marcelo Reyes-Eldblom, Francisco Moya-Torrecilla, Rafael Mondragon-Cortes, Daniel Rosado-Velazquez, Abel Gomez-Caceres

**Affiliations:** 1 Department of Physical Therapy, Faculty of Health Sciences, University of Malaga, 29071 Malaga, Spain; imp@uma.es; 2 Medical Services, Malaga Football Club, 29011 Malaga, Spain; rafamondragoncortes@gmail.com; 3 Onco-Hematology Unit, University Hospital Virgen de la Victoria, 29010 Malaga, Spain; martingarciapablo@gmail.com; 4 Puerperium Unit, Regional Hospital of Malaga, 29011 Malaga, Spain; sofiasanzdediego@gmail.com; 5 Costa del Sol Medical Center, 29620 Torremolinos, Spain; mredlblom@gmail.com; 6 Vithas Xanit International Hospital, 29630 Benalmadena, Spain; franmoya57@hotmail.com; 7 Medical Services, Real Madrid Football Club, 28055 Madrid, Spain; dr_rosado2002@hotmail.com; 8 HM Hospitals, 29010 Malaga, Spain; gomezcaceres@gmail.com

**Keywords:** growth factors, autologous platelet-rich plasma, injection, ankle instability, recurrent ankle sprain, lateral ligament injury, chronic injury

## Abstract

The platelet-rich plasma (PRP) approach may be an effective treatment for joint and cartilage pathologies. However, the rationale for its effectiveness on joint instability is limited. This study aimed to assess the safety and effectiveness of PRP injections in patients with chronic lateral ankle instability (CLAI). This retrospective study was performed at a single-center outpatient clinic between January 2015 and February 2023 and included pre-intervention assessment and short-term follow-up. Patients were excluded if they had received previous surgical treatment or had constitutional hyperlaxity, systemic diseases, or grade II or III osteoarthritis. The clinical and functional evaluation consisted of the Karlsson score, the Cumberland Ankle Instability Tool (CAIT), Good’s grading system, the patient’s subjective satisfaction level, and the time required to return to exercise. The entire PRP therapy regime consisted of three PRP administrations at 7-day intervals and follow-up appointments. PRP was administered both intraarticularly and into talofibular ligaments. A total of 47 consecutive patients with CLAI were included, 11 were female (23.4%), with a mean age at intervention of 31.19 ± 9.74 years. A statistically significant improvement was found in the CAIT and Karlsson scores at 3 months (27.74 ± 1.68 and 96.45 ± 4.28, respectively) relative to the pre-intervention status (10.26 ± 4.33 and 42.26 ± 14.9, respectively, *p* < 0.000). The mean follow-up of patients with CLAI was 17.94 ± 3.25 weeks. This study represents successful short-term functional and clinical outcomes in patients with CLAI after PRP treatment, with no adverse effects. It demonstrates the feasibility of a randomized controlled trial to further assess this therapy.

## 1. Introduction

Chronic lateral ankle instability (CLAI) is an acquired musculoskeletal condition that has become an issue of interest among health care providers and sports population due to its high prevalence [1]. Symptoms normally include persistent discomfort or tenderness and swelling; a wobbly or unstable sensation in the ankle; and the repeated turning of the ankle, especially on uneven surfaces or when participating in sports [2,3]. The anterolateral recess of the tibiotalar joint has been demonstrated to be the anatomical area mainly involved in painful sensation in patients with CLAI. This is a virtual space between the lateral articular facet of the talus and the undersurface of the ATFL, which is secondarily affected by a phenomenon of injury/overuse of this ligament [4]. Current conservative approaches to CLAI include oral medication, physiotherapy, strengthening and balance programs, and orthosis use. These methods are mainly focused on relieving symptoms and compensating passive stabilization deficits [5]. To date, no definite, effective therapy has been introduced, leading to chronic use of ankle orthosis or surgery. Although sometimes partially effective, these therapies tend not to provide patients with the desired results, and surgery often becomes the last algorithm-based step. Thus, new therapies providing functional and clinical improvements are needed.

In the last two decades, promising results have been obtained on articular pathology based on regenerative therapies through autologous preparations [6,7,8,9]. Platelet-rich plasma (PRP) regimen injections are nowadays being used as an alternative for treating musculoskeletal conditions that have failed to be managed by conservative management and prior to surgical options [10]. PRP has emerged as a safe, cost-effective procedure with additional benefits that involves minimal risk [11]. Despite positive results on soft-tissue and degenerative pathologies, there is currently limited evidence supporting the use of PRP in treating ankle ligament pathologies [12]. According to its nature and mechanism of action, it is becoming increasingly apparent that this therapy can reduce the severity of the injury and might improve the return to activity in CLAI patients. Therefore, the specific purpose of the current case series was to document the effectiveness of multiple PRP injections in ameliorating functional impairment and reducing symptoms and re-sprains associated with CLAI in a realistic clinical setting.

## 2. Materials and Methods

This is a retrospective review of prospectively assessed data of CLAI cases treated in a single-center outpatient clinic between January 2015 and February 2023. Patients were referred from different outpatient orthopedic surgeons with the diagnosis of CLAI. The following inclusion criteria were considered: (1) aged >18 years; (2) a history of previous unilateral ankle sprain as relevant trauma and presenting pain spontaneously and upon palpating the lateral side of the ankle; (3) previously failed non-operative management (bracing, taping, and physical therapy); (4) a CAIT score of ≤25 [13]; (5) reporting instability sensations and failure during daily and/or sports activities; (6) no use of physical therapy or changes in shoes or orthotic devices during the study period; (7) positive results on anterior drawer and tilt test on physical examination. Exclusion criteria were previous surgery in the ankle/foot region, corticosteroid injection in the affected region within the last month, presence of open wounds or skin diseases in the area to be treated, systemic or local infection, coagulation disorders/prolongation of bleeding time (e.g., those receiving anticoagulant drug therapy), known or suspected joint infection, fracture in either lower extremity requiring realignment, constitutional hyperlaxity, and rheumatological disorders. Additionally, patients with local infection at the site of the procedure, consistent use of nonsteroidal anti-inflammatory drugs within 2 days of procedure, and systemic corticosteroid intake within 1 month were excluded. Patients were also excluded if they had received previous surgical treatment or had constitutional hyperlaxity, systemic diseases, or grade II or III osteoarthritis.

### 2.1. Ethics

The present study was carried out in accordance with the Declaration of Helsinki in its latest revised version [14] and Good Clinical Practice Regulations (International Conference for Harmonization) [15], with both being international standards on clinical trials. The study design was a retrospective case series and was approved by our institutional review board for research (protocol code No. CEI-MCF: 6-2022-H; date: 10 January 2022). All patients provided signed informed consent for participation in the study.

### 2.2. Study Population and Procedure

This study consisted of a chart review of patients found to have CLAI, whose demographic data, including type and level of physical activity, were evaluated. All involved patients underwent a screening visit, a baseline assessment visit, and a follow-up visit at 3 months. All participants were given PRP by a single operator and an assistant to aid in preparation, and maintenance of the aseptic technique was ensured during the entire process. The injections were performed by the same experienced practitioner, who did not participate in the clinical assessment or in the data analysis. The clinical assessment was documented by a single investigator for each patient prior to the injection series and at 3 months after the first injection. For the purposes of this investigation, CLAI was defined as recurrent sprains or repeated sense of “giving way” resulting from trauma for at least 12 months without adequate response to conservative treatment [1].

### 2.3. Outcome Measures

The primary outcome measure was the change in functional outcome over time comparing pre-intervention and short-term patient-reported outcome measures according to the CAIT and Karlsson scores [16]. As secondary outcome measure, the Visual Analogue Scale (VAS), the score by Good et al. [17], and the patient’s subjective global satisfaction level with the procedure were used at baseline and final follow-up. Good et al.’s grade was classified as excellent—grade I; good—grade II; fair—grade III; or poor—grade IV. When rated good or excellent, it was considered a satisfactory result in our study. Safety was assessed by recording the occurrence of local/systemic adverse events or complications reported spontaneously by the patient or observed by the investigator or their staff. Outcome data were collected at baseline and 3 months after intervention and were analyzed. Recurrent sprain or instability and return-to-preinjury-level rates were also recorded by means of a telephone interview conducted by independent investigators. Image assessment was not performed, as diagnostic imaging is not considered a gold-standard tool in CLAI.

**Figure 1:**
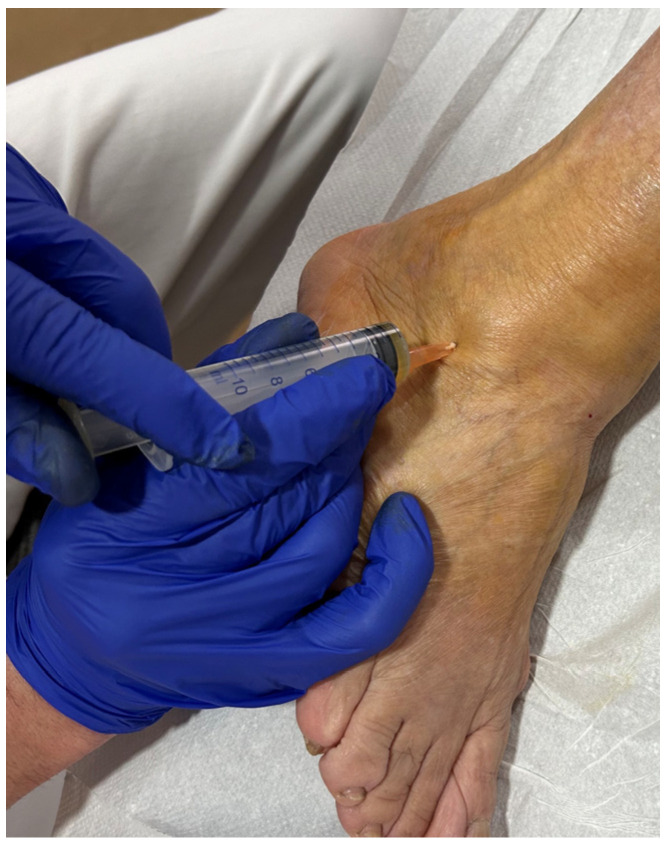
Injection of the anterior talofibular ligament.

A total of 54 mL of whole blood was aseptically collected from the medial cubital vein of every patient. Then, the blood underwent a standardized protocol of preparation, activation, and injection of the platelet concentrate, which has been described elsewhere [18]. This is considered P2-x-Bβ PRP according to the PAW classification, where P is for platelets, A is for activator, and W is for white cells [19]. Patients were placed in a supine position with knees extended and ankle positioned straight. PRP was injected intraarticularly through an anteromedial approach by using a 23-gauge needle, whereas a 25-gauge needle was inserted adjacent to and into talofibular ligaments (Figure 1). Injection points were determined by identifying anatomical landmarks based on previously described approaches. All procedures were performed by following this guideline and under sterile conditions [20]. Each patient received a set of three PRP injections into the ankle joint and talofibular ligaments at seven-day intervals over two weeks. Each injection was performed by the same treating practitioner at a single institution.

Local anesthetic was not administered before injection in any case to prevent possible negative interactions due to its potential negative influence on PRP action through pH modifications [21]. In this sense, participants were also instructed to avoid the use of NSAIDs during treatment up to the follow-up visit. Post-injection instructions were discussed with the patients, including the application of local cryotherapy every 2 h for 15 min duration, the use of analgesic medication as a rescue therapy for post-interventional pain, and active-assisted and active range-of-motion exercises of the treated areas. Patients were also allowed to fully weight bear and to resume their daily living activities. However, they were advised to avoid impact activities such as running and jumping up to 2 weeks after the last PRP injection. A gradual increase in impact activities was recommended over the following 4–6 weeks. Return to sport was allowed based on individual progression and requirements.

### 2.4. Statistical Analysis

All data were assessed for normality by using the Kolmogorov–Smirnov test. The paired Student’s *t*-test was used for continuous variables to test for significant differences between baseline and follow-up measurements. The McNemar test was used for categorical variables to evaluate the significance of the association between the two moments. All statistical analyses were performed by using SPSS (Statistical Package of Social Sciences; Chicago, IL, USA) for Windows software program, version 25.0. A *p*-value of less than 0.05 was considered statistically significant. Due to the small sample size, dependencies in the data (*n* = 3 bilateral cases) were not considered in the statistical tests and were excluded for all purposes.

## 3. Results

A total of 52 potential participants were assessed for eligibility, of whom 49 fulfilled our inclusion criteria and consented to be part of the study. There were two withdrawals during the study period due to not returning for follow-up visits. A total of 47 patients completed the study, with 23.4% being female. The right ankle was involved in 30 patients and the left in 17, with an average recurrent sprain of 4.77. The mean duration of symptoms was 29.96 months (range of 6 to 96 months), and the mean follow-up time was 17.94 weeks (range of 12 to 25 weeks). The demographic and clinical characteristics of the patients are summarized in Table 1. A total of 23 patients (48.93%) were recreational athletes with a non-regulated physical activity, and 20 of them (42.55%) belonged to a sports club or were involved in an organized physical activity, while 4 (8.51%) were sedentary people. The sports activities and levels of activity for all study participants are outlined in Table 2.

### Clinical and Functional Outcomes

The CAIT score improved significantly from an average pre-intervention score of 10.26 (range of 0 to 24) to 27.74 (range of 26 to 30) at the follow-up (*p* < 0.00), whereas the Karlsson score changed from an average baseline score of 42.26 (range of 7 to 70) to 96.45 (range of 88 to 100) over the same follow-up period (*p* < 0.00) (Table 3). When accurately evaluating the pain scores, a statistically significant improvement was noted in the VAS, with a notable change in scores from 7.15 to 0.66 (*p* < 0.00). Based on Good’s grading system, the patient sample consisted of 46 Grade I—excellent—cases and 1 Grade II—good—cases at the final follow-up. Therefore, all cases (100%) achieved satisfactory functional results (Table 3). The patients’ subjective global satisfaction level with the procedure and outcomes was good/satisfied (27.7%) or excellent/very satisfied (72.3%) at the final follow-up (Table 4). Although three patients (6.38%) reported recurrent sprains, all of them stated that they were satisfied with the procedure.

No clinical complications were observed after the PRP procedure. However, patients did report different degrees of postinjection inflammation at the injection site, including localized mild swelling and joint heaviness, which was controlled by local ice application for the first 24/48 h after treatment. Two patients (4.25%) reported transient dysesthesias on the anterior aspect of the ankle, which resolved by the first week. No additional undesirable postprocedure consequences, such as infections, nerve irritations, neuropathies, or systemic adverse events, were reported. The overall return-to-self-activity rate was 100%, with 40 patients (85.11%) resuming individual physical activity at preinjury levels. The failure rate was 6.38%, with three patients reporting reduced residual instability after a traumatic event. In all cases, the event was secondary to traumatic inversion ankle mechanism during sports activities.

## 4. Discussion

The present retrospective study, to our knowledge, represents the first case series involving PRP-treated CLAI cohorts. Forty-seven patients were successfully treated with a set of three PRP injections at weekly intervals, with a mean follow-up time of nearly 18 weeks. Type and level of physical activity and return-to-self-activity were also assessed in our study, with no reported complications.

Evidence from the scientific literature suggests that the characteristics of populations affected by recurrent ankle injury are heterogeneous. As several attempts have been made to define this condition, such as recurrent ankle instability, functional ankle instability, mechanical ankle instability, and the abovementioned CLAI, the conception of the problem itself is controversial [22,23,24]. The fact that no current consensus on a treatment algorithm exists and with current evidence being insufficient to confirm the benefits of one treatment modality over another, the approach of CLAI also remains a challenge.

Over the last decades, the employment of PRP therapy has substantially gained interest as a therapeutic weapon for repairing and regenerating damaged soft tissues in the foot and ankle region. Its well-demonstrated pathophysiologic action has the potential to minimize and restore the inherent damage caused to the affected structures [25]. However, most of the clinical investigations supporting the use of PRP in this region have attempted to shed light on the benefits of treating patients with ankle osteoarthritis (AO), where the pathophysiology of cartilage matrix degradation and progressive joint remodeling play a key role. According to our proposal, the intraarticular approach might be the common aspect between both pathological entities. Recent animal studies have demonstrated enhanced ligament repair following PRP application by increasing cellularity and angiogenesis and promoting earlier and more organized ligament filling [26,27]. Additionally, complete tears of the ATFL have punctually shown the complete healing of the ligament and early ankle stabilization after PRP applications [28]. Though PRP use is being widely employed, there is a clear gap in the literature surrounding the efficacy of its use in CLAI patients. Hence, the aim of this study was to evaluate the efficacy and safety of the combined administration of PRP injections in patients with CLAI and previous failed non-operative management. This approach is intended to include both intraarticular and extraarticular structures as potential therapeutic targets, since both histological and sensorimotor deficits have been demonstrated to be present in these patients [3].

Similar previous research has investigated the efficacy of PRP therapy in ankle ligament pathology. Zhang et al. compared different types of injection methods into the tear site of the anterior talofibular ligament (ATFL) and immobilization with immobilization alone in patients with acute lateral ankle sprain (LAS). After treatment, a significant reduction in VAS score and American Orthopedic Foot and Ankle Score (AOFAS) was documented at 6- and 12-month follow-ups [29]. These findings are in line with those achieved by Samra et al., who reported a significant improvement in functional capacity—agility (*p* = 0.002) and vertical jump (*p* = 0.001)—and reduction in fear avoidance (*p* = 0.014) after a single ultrasound-guided PRP administration into the anterior inferior tibiofibular ligament (AITFL) in 10 Rugby Union players within 14 days of MRI-confirmed ankle syndesmosis injury. In this pilot study, a historical control group were treated conservatively with the same inclusion criteria, and a rehabilitation protocol was used for comparison [30]. Similarly, a randomized controlled trial by Blanco-Rivera et al. evaluated patients with first-time grade II LAS treated with rigid immobilization for ten days. The experimental group previously received an isolated application of PRP over the ATFL in this study. At 8 weeks, the PRP patients presented the highest reduction in VAS score and better AOFAS than the control group. However, at 24 weeks, a similar evolution was documented in both groups [31]. Oppositely, a double-blinded study conducted by Rowden et al. to compare the effectiveness of the ultrasound-guided treatment of acute LAS with local anesthetic versus standard saline injection showed no statistical difference between the groups in terms of clinical and functional scores—VAS pain score and Lower Extremity Functional Scale (LEFS) [32]—Potential reasons for that could be the nature of the employed medications or the shortness of the follow-up evaluations. Our series adds to this body of evidence by being, to our knowledge, the first reported series of PRP injections for the CLAI condition. Specifically, forty-seven patients who had previously not responded to at least 3 months of traditional conservative treatment, including physical therapy and orthoses, volunteered to be treated with PRP injections over three sessions each 1 week apart. They were then followed up at least 3 months after the last injection. Our findings include improved function, pain, and subjective well-being and indicate no adverse effects at the final follow-up evaluation.

The herein presented results preliminarily grant PRP a preponderant role in the treatment of patients suffering from CLAI. Moreover, it would be reasonable that similar-in-nature conditions, such as shoulder, wrist, or patellar instabilities, may benefit equally from the suggested approach. Synovial irritation has been demonstrated to be present in chronically unstable ankles due to permanent mechanical stress on the synovium. Animal studies have shown to effectively alleviate this synovitis through three PRP injections applied weekly [33]. However, although PRP has been widely employed for treating traumatic or surgically created ligament injuries, conflicting data on its cellular and molecular effects have been observed [34]. More high-quality studies are required based on basic science data for pursuing any causal relationship between the use of PRP in ligament injuries and investigating its potential for regeneration and healing.

The rationale for the effectiveness of PRP in CLAI may rely on the shrinking effect on the capsule due to the hypothetical progressive diffusion of PRP inside the joint cavity. Additionally, the combination of intraarticular injections of PRP with peri-ligament administration increases the range of PRP action on CLAI, reaching more joint structures involved in the target pathology. Although our results did not prove to biologically solve this condition, both clinical and functional outcomes may represent a promising choice in comparison with any other conservative measure. Therefore, CLAI patients treated with the described PRP regime could benefit from these achievements, avoiding the predicted surgery. It should be noted that classical conservative approaches have been demonstrated not to be effective in our series and that surgery had been already indicated in certain cases.

The importance of the addition of PRP extraarticular injection to the conventional intraarticular approach in patients with joint instability lies in the separate roles that both ligaments and capsule may play in the development of CLAI. Previous biomechanical studies have demonstrated that talofibular ligaments and the talocrural joint capsule make an independent contribution to talocrural joint stability [35]. The existence of these separate roles would justify in itself the integral approach herein presented. Moreover, common histopathological findings in patients with CLAI include synovitis/fibrosis inside the anterolateral recess of the tibiotalar joint and chondral lesions affecting the articular facet of the talus. We then hypothesized that through this approach, not only a physiological shrinkage of the joint but also a reparative action could be obtained. Finally, this could be an explanation for the spontaneous verbal testimonies obtained from the treated patients and confirmed by patient-reported-outcome instruments.

An inherent limitation of clinical PRP studies is that both the amount of platelets and the quantity of activated growth factors in the PRP injections were not specifically calculated. However, inferences can be made through previously published laboratory studies. According to this, PRP of type P2-x-Bβ (PAW classification) was employed in our series, with platelet collection being approximately double the concentration in blood and without leukocytes [19]. This has also demonstrated positive correlations between the number of platelets and growth factor production rate [36,37]. Recent research has recommended a platelet concentration < 5-fold blood platelet concentration and a total absence of white cells as the main features of the PRP final product regarding applications in joint degeneration disorders. On the contrary, the presence of leucocytes may activate the inflammatory cascade, and an exaggerated amount of platelets may inhibit PRP action on tissue regeneration [38]. Nonetheless, in our series, no cell counts were performed at the time of whole-blood extraction, and no quantification of platelet or white cell lineage concentrations specific to the device was appraised.

The frequency of PRP application has become another issue of debate since its introduction. Outcomes from studies employing several repeated injections every one or two weeks have shown to be superior to those obtained through a single injection [39,40]. Our work included PRP therapy applied on three different occasions at one-week intervals. Oppositely, Vilchez-Cavazos sustained in their review that no evidence exists to corroborate these differences and that further high-quality studies are needed to establish solid conclusions [41].

Ultrasound (US) guidance during injection procedures has become a useful tool, whose use has increased exponentially in recent years. Advocates of this method suggest that it can increase the accuracy of the correct placement of specific therapies, thereby maximizing the benefit of the targeted therapy, helping to minimize needle trauma, and adequately identifying neurovascular structures. In addition, real-time visual control influences patient experience by reducing procedural pain or enables clinicians to aspirate effusions by seeing the fluid potentially improving patient satisfaction [42]. On the other hand, many clinicians use a landmark-guided approach based on the palpation of anatomical landmarks to detect the correct location of their injections. To date, this is still considered effective and less expensive than guided injections in the sports medicine field [43]. While both methods are designed to deliver active substances locally, little consensus exists as to the most effective approach, and the evidence base for asserting the superiority of one method over the other is inconclusive. US-guided injections at the ankle and foot have proven superior efficacy to landmark-guided injections in cadaveric studies [44]. However, several reviews have been unable to establish any advantage in terms of pain and function of US-guided injection for different musculoskeletal disorders over landmark-guided approaches [43,45,46]. The lack of US guidance in our series may have played a role and interfered negatively with identifying neural structures or inoculating PRP at undesired sites. The precise placement of PRP product results in higher odds for the required regenerating action. A blinded approach to identifying anatomical landmarks based on previously published guidelines was employed in our series [20]. This might have played a role in those two cases with postinjection transient dysesthesia, and identifying the involved nerve branch could have been possible.

This study has certain limitations that are noteworthy. First, methodological limitations include a small sample size and a relatively short follow-up period. Additionally, a control group was not included, and the wide age range and physical activity levels were potential sources of heterogeneity. For these reasons, a chance may exist that we did not have enough power to detect improvement in some of the PROs. Therefore, larger randomized controlled trials with more homogenous samples should be performed before PRP can be included in clinical routine. Second, the observed clinical and functional improvement after PRP injections in our series could have been in part due to the natural course of the condition or the adjunct non-operative management; however, this positive natural course has not been documented in the past, and the referred conservative therapy was applied before injection in all cases. Therefore, it is unlikely that both variables would have provided additional benefits separately or in combination. Third, the existence of a wide variety of manufacturers and PRP preparations might interfere with the extrapolation of our results to all available PRP interventions. Beyond these limitations, this study provided preliminary insights into the therapeutic benefits and limits of PRP therapy in CLAI.

## 5. Conclusions

PRP is being recognized as an appropriate method for the treatment of musculoskeletal injuries. While PRP is a very exciting field of research, a fair number of questions still need answers. Optimal parameters for preparation and administration, along with dosage, frequency, and number of injections, are still a matter of debate. Despite this, our results show that PRP injections represent a safe and effective method for CLAI patients, improving symptoms and function, creating no adverse effects, and lasting for at least 3 months. Larger high-quality studies are needed to evaluate risks and benefits of PRP in CLAI patients.

## Figures and Tables

**Table 1:** Patient demographics. Values are expressed as means ± standard deviations.

Variable	Value
Patients eligible for the study, No.	52
Patients lost to follow-up, No. (%)	5
Patients included in the study, No.	47
Gender (male/female), No.	36/11
Age at the time of intervention	31.19 ± 9.74
Duration of symptoms, mean (range), mo	29.96 ± 19.71
Follow-up, mean (range), mo	17.94 ± 3.25
Affected ankle (right/left), No.	30/17
No. of sprains	4.77 ± 1.54

Values are expressed as means ± standard deviations.

**Table 2:** Sports activities and levels of activity of the sample.

Sports Activity	N (%)
Soccer	14 (29.78)
Sedentary	4 (8.51)
Indoor soccer	3 (6.38)
Handball	2 (4.25)
Basketball	1 (2.13)
Running	9 (19.15)
Ballet	2 (4.25)
Martial arts	1 (2.13)
Horse riding	2 (4.25)
Gym activities	9 (19.15)

**Table 3:** Clinical and functional outcomes after PRP regimen.

Outcome Measure	Baseline	Final Follow-Up	*p*-Value
Karlsson score (0–100)	42.26 ± 14.9	96.45 ± 4.28	0.00
CAIT (0–30)	10.26 ± 4.33	27.74 ± 1.68	0.00
Pain—VAS (0–10)	7.15 ± 1.04	0.66 ± 0.78	0.00

Values are expressed as means ± standard deviations. CAIT, Cumberland Ankle Instability Tool; VAS, Visual Analogue Scale.

**Table 4:** Evaluation of functional results (Good’s grading system) and global satisfaction level.

		No. of Patients (N = 47)
Global satisfaction level	Very satisfied	34
	Satisfied	13
	Acceptable	-
	Unsatisfied	-
Good’s grading system	Grade 1—Full activity, including strenuous sport. No pain, swelling, or giving way.	46
	Grade 2—Occasional aching only following strenuous exercise. No giving way or feeling of apprehension.	1
	Grade 3—No giving way but some remaining apprehension and takes care when walking on rough ground.	-
	Grade 4—Recurrent instability and giving way in normal activities, with episodes of pain and swelling.	-

## Data Availability

Data are contained within the article.

## References

[1] Gribble P.A., Delahunt E., Bleakley C., Caulfield B., Docherty C.L., Fourchet F., Fong D., Hertel J., Hiller C., Kaminski T.W. (2013). Selection Criteria for Patients with Chronic Ankle Instability in Controlled Research: A Position Statement of the International Ankle Consortium. J. Orthop. Sports Phys. Ther..

[2] Yu P., Cen X., Mei Q., Wang A., Gu Y., Fernandez J. (2024). Differences in Intra-Foot Movement Strategies during Locomotive Tasks among Chronic Ankle Instability, Copers and Healthy Individuals. J. Biomech..

[3] Wenning M., Gehring D., Mauch M., Schmal H., Ritzmann R., Paul J. (2020). Functional Deficits in Chronic Mechanical Ankle Instability. J. Orthop. Surg..

[4] Ricci V., Mezian K., Chang K.-V., Onishi K., Kara M., Naňka O., Özçakar L. (2024). Ultrasound-Guided Injection of the Ankle Joint: Cadaveric Investigation of the Anterolateral Approach. Surg. Radiol. Anat. SRA.

[5] Hong C.C., Tan K.J., Calder J. (2024). Chronic Lateral Ankle Ligament Instability—Current Evidence and Recent Management Advances. J. Clin. Orthop. Trauma.

[6] Haddad C., Zoghbi A., El Skaff E., Touma J. (2023). Platelet-Rich Plasma Injections for the Treatment of Temporomandibular Joint Disorders: A Systematic Review. J. Oral Rehabil..

[7] Goodwin B., Averell N., Al-Shehab U., Ernazarov A., Price L., Choudhary A., Jermyn R. (2023). Efficacy of Platelet-Rich Plasma for Sacroiliac Joint Dysfunction: A Qualitative Systematic Review with Pooled Analysis. Regen. Med..

[8] Wu J., Du Z., Lv Y., Zhang J., Xiong W., Wang R., Liu R., Zhang G., Liu Q. (2016). A New Technique for the Treatment of Lumbar Facet Joint Syndrome Using Intra-Articular Injection with Autologous Platelet Rich Plasma. Pain Physician.

[9] Bennell K.L., Hunter D.J., Paterson K.L. (2017). Platelet-Rich Plasma for the Management of Hip and Knee Osteoarthritis. Curr. Rheumatol. Rep..

[10] Mlynarek R.A., Kuhn A.W., Bedi A. (2016). Platelet-Rich Plasma (PRP) in Orthopedic Sports Medicine. Am. J. Orthop..

[11] Raeissadat S.A., Rahimi M., Rayegani S.M., Moradi N. (2023). Cost-Utility Analysis and Net Monetary Benefit of Platelet Rich Plasma (PRP), Intra-Articular Injections in Compared to Plasma Rich in Growth Factors (PRGF), Hyaluronic Acid (HA) and Ozone in Knee Osteoarthritis in Iran. BMC Musculoskelet. Disord..

[12] Arthur Vithran D.T., He M., Xie W., Essien A.E., Opoku M., Li Y. (2023). Advances in the Clinical Application of Platelet-Rich 362 Plasma in the Foot and Ankle: A Review. J. Clin. Med..

[13] Wright C.J., Arnold B.L., Ross S.E., Linens S.W. (2014). Recalibration and Validation of the Cumberland Ankle Instability Tool Cutoff Score for Individuals with Chronic Ankle Instability. Arch. Phys. Med. Rehabil..

[14] General Assembly of the World Medical Association (2014). World Medical Association Declaration of Helsinki: Ethical Principles for Medical Research Involving Human Subjects. J. Am. Coll. Dent..

[15] Dixon J.R. (1998). The International Conference on Harmonization Good Clinical Practice Guideline. Qual. Assur..

[16] Houston M.N., Hoch J.M., Hoch M.C. (2015). Patient-Reported Outcome Measures in Individuals with Chronic Ankle Instability: A Systematic Review. J. Athl. Train..

[17] Good C.J., Jones M.A., Lingstone B.N. (1975). Reconstruction of the Lateral Ligament of the Ankle. Injury.

[18] Fiz N., Delgado D., Garate A., Sánchez P., Oraa J., Bilbao A.M., Guadilla J., Sánchez M. (2020). Intraosseous Infiltrations of Platelet-Rich Plasma for Severe Hip Osteoarthritis: A Pilot Study. J. Clin. Orthop. Trauma.

[19] DeLong J.M., Russell R.P., Mazzocca A.D. (2012). Platelet-Rich Plasma: The PAW Classification System. Arthrosc. J. Arthrosc. Relat. Surg. Off. Publ. Arthrosc. Assoc. N. Am. Int. Arthrosc. Assoc..

[20] de Cesar Netto C., da Fonseca L.F., Simeone Nascimento F., O’Daley A.E., Tan E.W., Dein E.J., Godoy-Santos A.L., Schon L.C. (2018). Diagnostic and Therapeutic Injections of the Foot and Ankle—An Overview. Foot Ankle Surg. Off. J. Eur. Soc. Foot Ankle Surg..

[21] Foster T.E., Puskas B.L., Mandelbaum B.R., Gerhardt M.B., Rodeo S.A. (2009). Platelet-Rich Plasma: From Basic Science to Clinical Applications. Am. J. Sports Med..

[22] Konradsen L., Bech L., Ehrenbjerg M., Nickelsen T. (2002). Seven Years Follow-up after Ankle Inversion Trauma. Scand. J. Med. Sci. Sports.

[23] Hertel J. (2002). Functional Anatomy, Pathomechanics, and Pathophysiology of Lateral Ankle Instability. J. Athl. Train..

[24] Delahunt E., Coughlan G.F., Caulfield B., Nightingale E.J., Lin C.-W.C., Hiller C.E. (2010). Inclusion Criteria When Investigating Insufficiencies in Chronic Ankle Instability. Med. Sci. Sports Exerc..

[25] Johnson L.G., Buck E.H., Anastasio A.T., Abar B., Fletcher A.N., Adams S.B. (2022). Efficacy of Platelet-Rich Plasma in Soft Tissue Foot and Ankle Pathology. JBJS Rev..

[26] Fleming B.C., Spindler K.P., Palmer M.P., Magarian E.M., Murray M.M. (2009). Collagen-Platelet Composites Improve the Biomechanical Properties of Healing Anterior Cruciate Ligament Grafts in a Porcine Model. Am. J. Sports Med..

[27] Prządka P., Kiełbowicz Z., Osiński B., Dzimira S., Madej J.A., Nowacki W., Kubiak K., Reichert P., Cegielski M. (2017). Reconstruction of Cranial Cruciate Ligament in Rabbits Using Polyester Implants Saturated with PRP, Antlerogenic Stem Cells MIC-1 and Their Homogenate. Connect. Tissue Res..

[28] Lai M.W.W., Sit R.W.S. (2018). Healing of Complete Tear of the Anterior Talofibular Ligament and Early Ankle Stabilization after Autologous Platelet Rich Plasma: A Case Report and Literature Review. Arch. Bone Jt. Surg..

[29] Zhang J., Wang C., Li X., Fu S., Gu W., Shi Z. (2022). Platelet-Rich Plasma, a Biomaterial, for the Treatment of Anterior Talofibular Ligament in Lateral Ankle Sprain. Front. Bioeng. Biotechnol..

[30] Samra D.J., Sman A.D., Rae K., Linklater J., Refshauge K.M., Hiller C.E. (2015). Effectiveness of a Single Platelet-Rich Plasma Injection to Promote Recovery in Rugby Players with Ankle Syndesmosis Injury. BMJ Open Sport Exerc. Med..

[31] Blanco-Rivera J., Elizondo-Rodríguez J., Simental-Mendía M., Vilchez-Cavazos F., Peña-Martínez V.M., Acosta-Olivo C. (2020). Treatment of Lateral Ankle Sprain with Platelet-Rich Plasma: A Randomized Clinical Study. Foot Ankle Surg. Off. J. Eur. Soc. Foot Ankle Surg..

[32] Rowden A., Dominici P., D’Orazio J., Manur R., Deitch K., Simpson S., Kowalski M.J., Salzman M., Ngu D. (2015). Double-Blind, Randomized, Placebo-Controlled Study Evaluating the Use of Platelet-Rich Plasma Therapy (PRP) for Acute Ankle Sprains in the Emergency Department. J. Emerg. Med..

[33] Yin J., Xu Z., Liu J. (2020). Alleviation of Synovitis Caused by Joint Instability with Application of Platelet-Rich Plasma. Thromb. Res..

[34] Kunze K.N., Pakanati J.J., Vadhera A.S., Polce E.M., Williams B.T., Parvaresh K.C., Chahla J. (2022). The Efficacy of Platelet-Rich Plasma for Ligament Injuries: A Systematic Review of Basic Science Literature with Protocol Quality Assessment. Orthop. J. Sports Med..

[35] Li L., Gollhofer A., Lohrer H., Dorn-Lange N., Bonsignore G., Gehring D. (2019). Function of Ankle Ligaments for Subtalar and Talocrural Joint Stability during an Inversion Movement—An In Vitro Study. J. Foot Ankle Res..

[36] Mishra A., Woodall J., Vieira A. (2009). Treatment of Tendon and Muscle Using Platelet-Rich Plasma. Clin. Sports Med..

[37] Eppley B.L., Woodell J.E., Higgins J. (2004). Platelet Quantification and Growth Factor Analysis from Platelet-Rich Plasma: Implications for Wound Healing. Plast. Reconstr. Surg..

[38] Milants C., Bruyère O., Kaux J.-F. (2017). Responders to Platelet-Rich Plasma in Osteoarthritis: A Technical Analysis. BioMed Res. Int..

[39] Kavadar G., Demircioglu D.T., Celik M.Y., Emre T.Y. (2015). Effectiveness of Platelet-Rich Plasma in the Treatment of Moderate Knee Osteoarthritis: A Randomized Prospective Study. J. Phys. Ther. Sci..

[40] Tavassoli M., Janmohammadi N., Hosseini A., Khafri S., Esmaeilnejad-Ganji S.M. (2019). Single- and Double-Dose of Platelet-Rich Plasma versus Hyaluronic Acid for Treatment of Knee Osteoarthritis: A Randomized Controlled Trial. World J. Orthop..

[41] Vilchez-Cavazos F., Millán-Alanís J.M., Blázquez-Saldaña J., Álvarez-Villalobos N., Peña-Martínez V.M., Acosta-Olivo C.A., Simental-Mendía M. (2019). Comparison of the Clinical Effectiveness of Single Versus Multiple Injections of Platelet-Rich Plasma in the Treatment of Knee Osteoarthritis: A Systematic Review and Meta-Analysis. Orthop. J. Sports Med..

[42] Soh E., Li W., Ong K.O., Chen W., Bautista D. (2011). Image-Guided versus Blind Corticosteroid Injections in Adults with Shoulder Pain: A Systematic Review. BMC Musculoskelet. Disord..

[43] Daniels E.W., Cole D., Jacobs B., Phillips S.F. (2018). Existing Evidence on Ultrasound-Guided Injections in Sports Medicine. Orthop. J. Sports Med..

[44] Khosla S., Thiele R., Baumhauer J.F. (2009). Ultrasound Guidance for Intra-Articular Injections of the Foot and Ankle. Foot Ankle Int..

[45] Saha P., Smith M., Hasan K. (2023). Accuracy of Intraarticular Injections: Blind vs. Image Guided Techniques-A Review of Literature. J. Funct. Morphol. Kinesiol..

[46] Bloom J.E., Rischin A., Johnston R.V., Buchbinder R. (2012). Image-Guided versus Blind Glucocorticoid Injection for Shoulder Pain. Cochrane Database Syst. Rev..

